# Cholinergic Modulation Promotes Attentional Modulation in Primary Visual Cortex- A Modeling Study

**DOI:** 10.1038/s41598-019-56608-3

**Published:** 2019-12-27

**Authors:** Atena Sajedin, Mohammad Bagher Menhaj, Abdol-Hossein Vahabie, Stefano Panzeri, Hossein Esteky

**Affiliations:** 1 Department of Electrical Engineering, Amirkabir University of Technology, Hafez Ave., 15875-4413 Tehran, Iran; 2 School of Cognitive Sciences (SCS), Institute for Research in Fundamental Sciences (IPM), 19395-5746 Tehran, Iran; 3 Neural Computation Laboratory, Center for Neuroscience and Cognitive Systems @UniTn, Istituto Italiano di Tecnologia, 38068 Rovereto, Italy; 4 Research Group for Brain and Cognitive Sciences, School of Medicine, Shahid Beheshti Medical University, 19839-63113 Tehran, Iran

**Keywords:** Cognitive neuroscience, Computational neuroscience

## Abstract

Attention greatly influences sensory neural processing by enhancing firing rates of neurons that represent the attended stimuli and by modulating their tuning properties. The cholinergic system is believed to partly mediate the attention contingent improvement of cortical processing by influencing neuronal excitability, synaptic transmission and neural network characteristics. Here, we used a biophysically based model to investigate the mechanisms by which cholinergic system influences sensory information processing in the primary visual cortex (V1) layer 4C. The physiological properties and architectures of our model were inspired by experimental data and include feed-forward input from dorsal lateral geniculate nucleus that sets up orientation preference in V1 neural responses. When including a cholinergic drive, we found significant sharpening in orientation selectivity, desynchronization of LFP gamma power and spike-field coherence, decreased response variability and correlation reduction mostly by influencing intracortical interactions and by increasing inhibitory drive. Our results indicated that these effects emerged due to changes specific to the behavior of the inhibitory neurons. The behavior of our model closely resembles the effects of attention on neural activities in monkey V1. Our model suggests precise mechanisms through which cholinergic modulation may mediate the effects of attention in the visual cortex.

## Introduction

Attention organizes our overwhelming sensory world and improves our ability to detect and discriminate behaviorally relevant sensory information^1–5^. Attentional modulation can enhance firing rates of cortical neurons^6–8^, influence their tuning properties^1,9^ and change receptive field boundary and size^8^. There is extensive experimental evidence for the involvement of acetylcholine (ACh) in attentional modulation^10–14^. Fronto-parietal axis plays a major role in attentional modulation of cortical sensory areas^15,16^ through direct cortico–cortical feedback connections and activation of cholinergic neurons in the basal forebrain corticocepetal system^12,17^. This forebrain nucleus represents the most rostral of the neuromodulatory cortical input systems and projects to sensory cortical areas. ACh, as a central nervous system neuromodulator, plays an essential role in many aspects of cognitive functions such as attention, arousal, vigilance, memory and learning^10,18,19^. At the cellular level, ACh can enhance the synaptic efficacy of thalamo-cortical connections through nicotinic receptors^20^ and suppress the synaptic efficacy of intra-cortical connections via muscarinic receptors^20^. Activation of muscarinic receptors results in improved neuronal excitability and increased inhibitory drive and aids attentional processing^10^.

ACh significantly enhances attentional modulation in the primary visual (V1) cortex neurons^10^. Depletion of ACh by disrupting cholinergic fibers results in diminished impact of attentional input^12^. In area V1 of anesthetized cats, ACh application increases firing rates and neuronal orientation tuning^21,22^. It also improves neuronal tuning and coding in area V1 of anesthetized monkeys^23^. In addition, ACh application imitates attentional effects on spatial integration and surround mechanisms in area V4^1,8^ and V1^9^. However, it should be noted that some studies have failed to replicate the effect of ACh on tuning curve sharpening and have reported diverse, and in some cases, contradictory results in V1 of anesthetized animals^24,25^.

It has been suggested that cortical orientation tuning profile of V1 neurons are mediated by activation of thalamocortical connections and by the contribution of inhibitory intracortical connections^26,27^. Cholinergic modulation controls synaptic efficacy of the thalamocortical and intracortical connections^20,28^. It increases inhibitory drive which can cause changes in the neural response selectivity similar to the effects of attention^13,29,30^. In addition, attention leads to a marked reduction in neuronal response variability of V1^31^ and V4^32^. Similar effects have been observed in rat V1 after basal forebrain stimulation^17,33^. Furthermore, attention decreases noise correlations of neurons in macaque area V1^31^ and V4^32,34,35^ and improve signal-to-noise ratio^32,35^. Similar effects are observed by increasing cholinergic drive in rat visual cortex in which ACh reduces noise correlation and enhances neural coding efficiency^33^. At network level, ACh has a significant control over the emergence of rhythmic activity in the cortical circuits^13^ and for synchronizing neuronal activity^28,36^. Importantly, attention enhances the synchrony of neuronal populations in V4^37^ and decreases the degree of neuronal gamma (30–50 Hz) synchrony in V1^38–40^.

While it is well established that ACh enhances visual sensory encoding, the precise mechanisms by which these effects are implemented at the cellular and circuit levels remain poorly understood. To study the mechanism through which ACh exerts its effect on sensory processing, we used a biophysically based recurrent V1 cortical network model to study the impact of cholinergic modulation on V1 neurons’ orientation tuning, spontaneous activity, noise correlation, neural response variability and gamma synchronization. The proposed model is a spiking neuronal network with a ring model structure^41,42^. We used the ring model structure as it replicates macaque V1 circuitries. We found that cholinergic modulation increased neural firing rates and sharpened the orientation tuning curves in network model layer 4C. Larger changes of the width of orientation tuning curve was observed when cholinergic input was introduced at high baseline activity conditions. In addition, ACh reduced noise correlations and neural response variability. These changes improved neural population coding accuracy. Importantly, ACh application resulted in a significant reduction in the magnitude of local field potential (LFP) oscillations and the spike-field coherence (SFC) in the gamma frequency range (30–50 Hz). Furthermore, ACh induced a highly desynchronized neural network state as indicated by the field potential activity of the neuronal populations while increasing the neural firing rates.

## Results

Here we studied how cholinergic modulation affects the response properties of a biologically inspired primary visual cortex (V1) network model. We used a V1 recurrent spiking ring network model with 1000 excitatory and 250 inhibitory neurons. The excitatory neurons were pooled into two groups (S1 and S2) each representing a separate location in space and only one of them, (S1), received the stimulus related external inputs (see Methods for details). Neurons were characterized by their spiking activities and received feed-forward input from the lateral geniculate nucleus (LGN) and lateral corticocortical inputs via excitatory to excitatory weights with cosine profile as indicated by experimental studies of the cholinergic effects in V1^43^. We configure a special structure into our model through coupling the preferred orientation of each neuron to its location in the network layer. Also, to sharpen the orientation selectivity of the units, recurrent cortical connections were set up in the network. We modeled two levels of ACh (see Methods, Data Analysis) to replicate the ACh concentration dependent effects of attention reported by experimental studies^44,45^.

### Effects of acetylcholine on orientation-tuning curves

**Figure 1:**
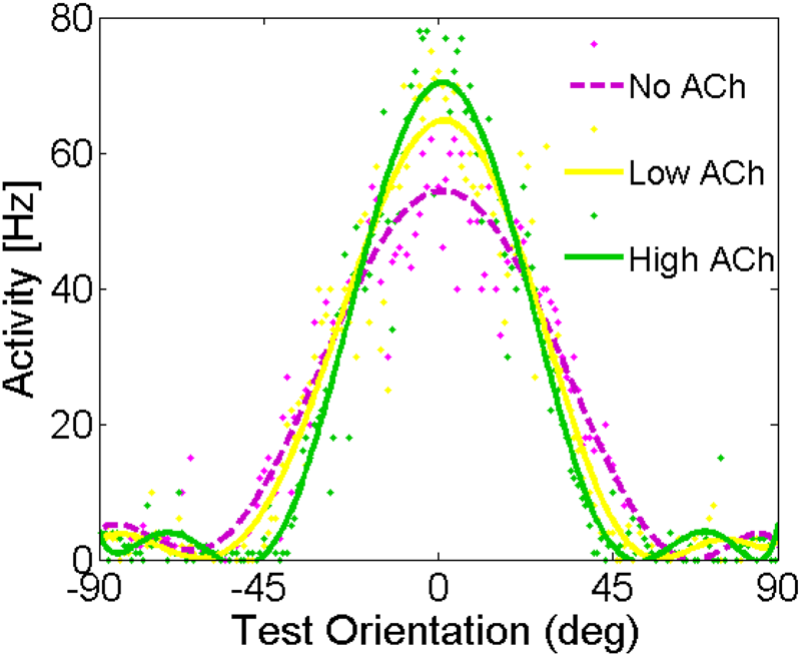
Model Behavior: Cholinergic Modulation Cause a Sharpening of Neural Tuning Curves. The network architecture consists of a recurrent network in which neurons receive feedforward input from the LGN and lateral input with excitation and inhibition (Mexican hat profile). Periodic boundary conditions (not shown) ensured a ring topology of the network. Tuning curve properties for the different concentration of ACh: Individual neuron tuning curves before and after applying ACh. The tuning curves are shown for neurons with a preferred orientation of zero after applying ACh.

First, we investigated how different concentrations of ACh affected V1 single neurons’ orientation tuning curves. In homogeneous ring neural networks, a neuron’s response before applying acetylcholine is a function of a single variable: the difference between the stimulus orientation *θ*_*stimulus* and the neuron’s preferred orientation *θ*. Cholinergic modulation rendered the network model inhomogeneous: the neural responses were characterized by two orientations, which are the orientation of the test stimulus and the preferred orientation of each neuron. Note that, each neuron’s preferred orientation refers to its preferred orientation before applying ACh. While our simulation indicated no difference in the preferred orientation before and after ACh administration the orientation tuning was significantly changed by cholinergic activation (Fig. 1). The amplitude of the fitted function for both concentrations of ACh was greater compared to the baseline in the tuning curves of the illustrated sample neuron. Smaller orientation tuning width, quantified as the half-width at half-height of the Gaussian functions, were observed for both concentrations of ACh. Specifically, in the presence of ACh, the orientation tuning widths were sharpened by 19.9% for high ACh level condition and 12.7% for low ACh level (Fig. 1; No ACh: 29.48 ± 0.11 deg, Low ACh: 26.14 ± 0.07 deg, and High ACh: 24.57 ± 0.07 deg). Sharpening of orientation tuning compared to the baseline was significant in the both ACh conditions (Wilcoxon signed rank test; *P* < 0.01).

### Effects of acetylcholine on LFPs and spike-triggered average (STA)

**Figure 2:**
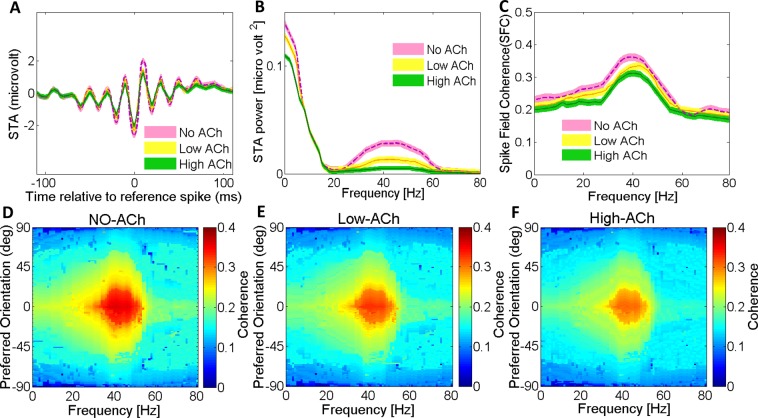
Cholinergic Modulation Reduces Spike-Triggered Average LFP Response and Spike-Field Gamma Coherence. To measure the synchronization between spikes and the local field potential (LFP), the spike-triggered average (STA) and its power spectrum are used: (**A**) ACh reduced the STA LFP response (compare green line versus yellow and dashed purple lines). (**B**) Power spectrum calculated from the STA LFP response in top. ACh strongly reduced the STA LFP power across most frequencies shown; this was most pronounced in the gamma frequency band. (**C**) ACh resulted in lower SFC (green and yellow lines) compared with that resulting from No-ACh condition (dashed purple line) at 0 degree orientation preference. Shaded areas, SEM. (**D**) ACh strongly decreased SFC in the gamma range. Applying low level of ACh (**E**) and applying high level of ACh (**F**) resulted in lower SFC compared with that resulting from control condition (**D**). Color bar indicates coherence values.

The regularity and amplitude of the local field potentials (LFP) fluctuations is a measure of synchronization of neural activity. Consequently, changes in the neural population’s synchrony are highly reflected between spikes and the LFP response correlation functions. We used Spike-Triggered Average (STA) of LFPs to measure the correlation. This measure is highly sensitive in detecting local neuronal synchronization (see Methods for details). Figure 2A presents the STA LFP of the proposed network model when different concentrations of ACh were applied to the network model. The STA LFPs oscillate together while there is no change in the oscillation phase across different trials. The STA LFP in the network model without ACh was more strongly modulated in the gamma frequency range (30–50 Hz) than when ACh was applied (Fig. 2B). Consequently, the power spectrum showed a larger peak in the gamma frequency range (30–50 Hz) for the NO-ACh condition (*P* < 0.05, Wilcoxon sign rank test). Our data indicates that ACh has a significant modulatory control over the emergence of the gamma rhythmic activity. We observed the highest power at low frequencies with a peak at around gamma frequency range (30–50 Hz). The power of gamma and low-frequency oscillations were larger for the network without any ACh, compared with applying low and high concentrations of ACh. These differences were significant (*P* < 0.05 two-factor repeated measurement [RM] ANOVA, ACh vs. no application).

### Effects of acetylcholine on spike-field coherence (SFC)

We used spike-field coherence (SFC) to measure phase synchronization between spikes and LFP oscillations as a function of frequency. SFC was normalized for spike rate and LFP power spectra to take ranges from zero (for complete lack of synchronization) to 1 (for perfect phase synchronization). Our SFC analysis showed a peak in the gamma range (30–50 Hz) that was significantly larger for the NO-ACh condition (Fig. 2C; *P* < 0.01, Wilcoxon signed rank test). SFC was reduced with applying ACh in both concentrations (Fig. 2D–F). The gamma frequency SFC significantly decreased by %19 and %31% with low and high ACh applications, respectively (*P* < 0.001, two-factor RM ANOVA). The same was true for low-frequency SFC as it was reduced by %11 and %6 with low and high ACh applications, respectively (*P* < 0.001, two-factor RM ANOVA). Thus, applying ACh with both concentrations affected the power spectrum of the STA LFP in the similar way as the SFC.

### Effects of acetylcholine on noise correlations

**Figure 3:**
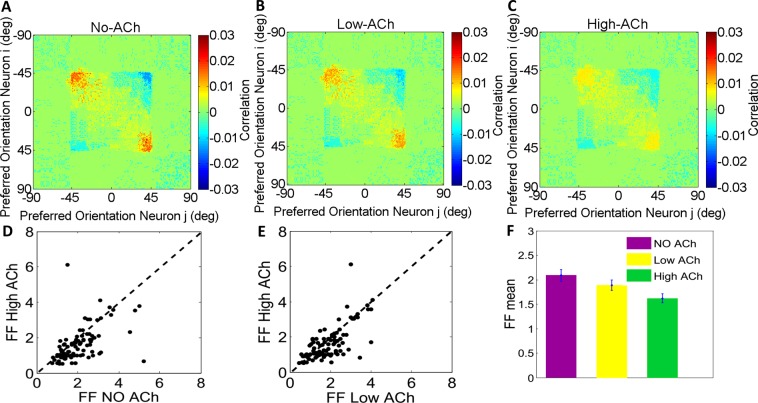
Cholinergic Modulation Induces Conspicuous Decorrelation and Enhances Response Reliability. ACh significantly decreased noise correlations. Spike count noise correlation is plotted for the pair of neurons and compared to the NO-ACh condition. (**A**) both concentrations (low & high) of applying ACh are significantly reduced noise correlations (**B** & **C**) respectively. The principal diagonal was omitted for clarity. (**D**) Fano factor (FF) distribution for administration of ACh and No-ACh condition. Measured at a bin size of 100 ms counting intervals spaced over the duration of the trial (filled circles). (**E**) As in (**D**) but distribution for administration of high level versus low level of ACh. (**F**) Bar graphs show mean of the respective distributions and SEM. The variability was significantly reduced when ACh was applied in different concentration compared to the control condition; p values indicate whether ACh application significantly affected FFs (two-factor ANOVA, ACh applied vs. not applied).

Noise correlation quantifies correlation of trial to trial response fluctuations to each stimulus. In our simulations, we modeled trial to trial activities as independent multiplicative noise to the firing rates of each neuron. This way we could model the Poisson-like variability that is reported in experimental studies^46,47^. We found that acetylcholine significantly enhanced the trial-to-trial response reliability of neurons (Fig. 3A–C; Wilcoxon signed rank test; *P* < 0.07).

Figure 3A–C depicts the noise correlation for 180 neurons each with a preferred orientation from −90 to+90 degrees responding to the stimulus orientation of zero degree. Only neurons with preferred orientations between around −45 and +45 degrees were activated by zero degree stimulus (the square section in the center of the matrix). These neurons indicate various degrees of correlation: the activity of neurons with similar preferred orientations, were highly correlated. But neurons with orientation tunings farther away from zero degree responded weakly to the stimulus and showed relatively negative correlations. This effect might be due to the fact that the noise changes the position of the activity bump in the network model but its shape is relatively kept constant. This stimulus falls at the edge of the RF of adjacent neurons. In these neurons the fluctuations in the position of the activity bump resulted in large and common rate fluctuations and consequently high correlations. Contrarily, the neurons at the center of the network population were poorly correlated since the exact position of the activity bump did not influence their firing rates.

### Effects of acetylcholine on rate variance

We characterized neuronal response reliability in the presence of acetylcholine by computing the Fano factor which is defined as the ratio of spike count variance to mean spike count (see Methods). We found that cholinergic activation in both concentrations caused significant decrease in the trial-to-trial response variability, as quantified by smaller Fano factor values during control and Acetylcholine-treated trials. We used two-factor ANOVA to calculate whether ACh significantly changed Fano factor values at population level (*P* < 0.001; Fig. 3D–F).

### Effects of acetylcholine concentration through different receptors on orientation tuning curves

**Figure 4:**
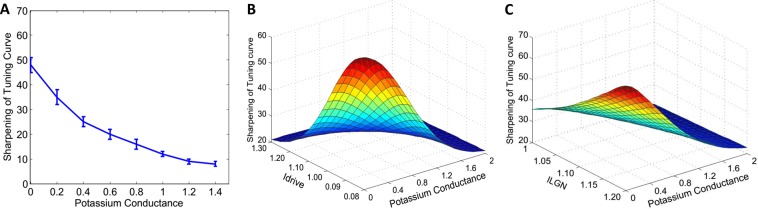
Interaction of Varying the Slow Potassium Conductance on Sharpening of the Tuning Curves and STA LFP Gamma Power. (**A**) Effects of varying the slow potassium conductance on sharpening of the tuning curves (half-width at half-height of the Gaussian functions). The error bars indicate the 95% confidence intervals. (**B**) Effects of varying the slow potassium conductance and Idrive on sharpening of the tuning curves. (**C**) Effects of varying the slow potassium conductance and ILGN on sharpening of the tuning curves. Averaged over 10 trials.

Little is known about synchronization and orientation selectivity of V1 neural network as a function of the degree of cholinergic activation. In our network, we modeled cholinergic effects by changing the conductance *g*_*KS*_ of slow potassium current that decreased as levels of acetylcholine increased, and *I*_*drive*_ an external current with Gaussian distribution across neurons by a variance set to result in one Hertz spread in the intrinsic neuronal frequencies for both low and high ACh concentrations. Figure 4A describes the dependence of sharpening of tuning curves upon *g*_*KS*_ in which *g*_*KS*_ is set to zero and 1.5 *ms*/*cm*^2^ to simulate high and low ACh concentrations. Sharpening of tuning curves significantly decreased with the increase of the potassium conductance (Wilcoxon signed rank test; *P* < 0.01). The STA LFP power in the gamma-frequency band increased significantly as the potassium conductance increased (Fig. S1; Wilcoxon signed rank test; *P* < 0.01). Varying the slow potassium conductance *g*_*KS*_ and *I*_*drive*_ on sharpening of the tuning curves showed that the sharpening of tuning curve strongly depended on the ACh activation (Fig. 4B). We also investigated nicotinic effects through perturbation thalamocortical input current *I*_*ffLGN*_ arriving from LGN to the network as shown experimentally^20,48,49^. Following muscarinic cholinergic modulations there was an increase in the neuronal firing rates and desynchronization of LFP gamma power and spike-field coherence (Fig. 4A,B and S1; Wilcoxon signed rank test; *P* < 0.01). But, we did not observe any systematic effects in orientation selectivity due to increased thalamocortical current (Fig. 4C; Wilcoxon signed rank test; *P* > 0.05).

### Effects of exclusive cholinergic activation of excitatory neurons

**Figure 5:**
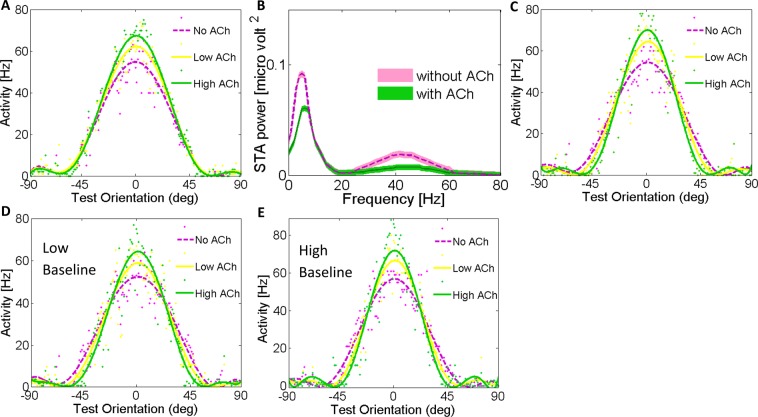
Acetylcholine Causes a Scaling of Neuronal Tuning Curves through administration to excitatory neurons And Comparison of Cholinergic Modulation between Two Excitatory pools S1 and S2 on the STA LFP Power Spectra. (**A**) Effects of applying ACh on the tuning curves of the individual neurons through administration to excitatory neurons, leads to significant sharpening without narrowing orientation tuning width. Tuning curve properties for the different concentration of ACh: Individual neuron tuning curves before and after applying ACh. The tuning curves are shown for neurons with a preferred orientation of zero after applying ACh. (**B**) The gamma modulation in without-ACh pool is much stronger than in with-ACh pool. ACh clearly desynchronize in the gamma-frequency band. Shaded areas s.e.m. (**C**) Applying ACh to 15% of the inhibitory interneurons, through muscarinic receptors, leads to significant sharpening (Wilcoxon signed rank test; P < 0.01). Effect of applying ACh on the tuning curves of the individual neurons, with different levels of baseline activity of the network. (**D**) Applying ACh with high baseline activity and (**E**) low baseline activity of the network, strongly alters orientation selectivity of V1 neurons.

When only excitatory neurons of our network were activated by ACh application, we observed a significant enhancement in the tuning amplitude by 26.1% (Fig. 5A; Wilcoxon signed rank test; *P* < 0.05). But there was no narrowing in the orientation tuning widths (Fig. 5A; *P* = 0.53). To further justify the cholinergic modulation effects in our model, we applied the cholinergic input to only one of the excitatory selective pools, and compared the effect of cholinergic modulation in two excitatory pools. The pool with ACh input showed increased firing rates and simultaneous STA and LFP power gamma rhythm reduction (Fig. 5B). The gamma modulation in the pool without ACh was up to 50% stronger than the pool with high level of ACh (S1). One possible reason could be that ACh reduces inhibitory surround mechanisms in the primary visual cortex similar to the effects of attention^39^. As a consequence, firing rates are enhanced, gamma oscillations are diminished simultaneously, and finally information processing are enhanced in the network model through activation of ACh receptors^28,33,36^.

### Effects of the size of inhibitory neural population that is activated by cholinergic activation on orientation-tuning curves

To test the degree of inhibitory neurons involvement in the observed findings we applied the cholinergic input to a smaller proportion of inhibitory neurons (%15 instead of %20). Interestingly, even in the low inhibitory condition there was a strong dependence of orientation selectivity and tuning width on the cholinergic modulation. However, the orientation tuning width depended strongly on the inhibitory drive. Tuning width, quantified as the half-width at half-height of the Gaussian function, for both levels of ACh narrowed. Specifically, applying ACh to 15% of the inhibitory interneurons in the population sharpened the orientation tuning width by 15.3% for high ACh level condition and 8% for low ACh level (Fig. 5C; No ACh: 29.48 ± 0.11 deg, Low ACh: 27.29 ± 0.12 deg, and High ACh: 25.55 ± 0.22 deg). Sharpening of orientation tuning compared to the baseline was significant in both ACh conditions (Wilcoxon signed rank test; *P* < 0.01). The higher the level of ACh the smaller the width and the sharper orientation selectivity. Thus, ACh modulation appears to scale the orientation tuning function. Noteworthy, we clearly show that the width of the orientation tuning curve decreases with increasing inhibition, and the inhibition may provide a significant threshold to sharpen the tuning width.

### Effects of cholinergic modulation and baseline activity alteration on the orientation-tuning curves

Here, we examined how cholinergic-dependent sharpening of orientation selectivity is affected by baseline activity changes in V1 model network. We modified the baseline activity in the network by increasing or decreasing the background external input by %20 percent.

In the high baseline activity condition cholinergic activation sharpened the orientation tuning width by 17.2% for high ACh level condition and 9.7% for low ACh level (Fig. 5D; No ACh: 27.98 ± 0.11 deg, Low ACh: 25.5 ± 0.1 deg, and High ACh: 23.87 ± 0.21 deg, Wilcoxon signed rank test; *P* < 0.01). Cholinergic activation at low baseline condition sharpened the orientation tuning width by 13.26% for high ACh level condition and 6% for low ACh level (Fig. 5E; No ACh: 31 ± 0.19 deg, Low ACh: 29.25 ± 0.15 deg, and High ACh: 27.37 ± 0.11 deg, Wilcoxon signed rank test; *P* < 0.01). Across the stimulations in network with high baseline, there was a 7% increase of orientation selectivity for high ACh (Fig. S2A), 7.1% for low ACh (Fig. S2B) and 5.3% for control condition (Fig. S2C) (Wilcoxon signed rank test; *P* < 0.005). On the contrary, in the low baseline activity of the network, there was a reduction of orientation selectivity by 7.12% (*P* < 0.005) for high ACh, 7.19% (*P* < 0.005) for low ACh, and 5.1% (*P* < 0.007) for control conditions (Fig. S2A–C). In high/low baseline activity in the presence of ACh yielded high/low orientation selective activity, respectively.

Our previous experimental studies in monkeys have shown that baseline activity affects encoding properties of visual neurons^50–52^. Here, to study the interaction between the impact of cholinergic input and baseline activity we applied ACh at low and high baseline activity conditions. Our results revealed that ACh application at high baseline condition results in larger improvement of the orientation selectivity compared with the low baseline condition.

### Effect of cholinergic modulation onset and duration on response synchronization

**Figure 6:**
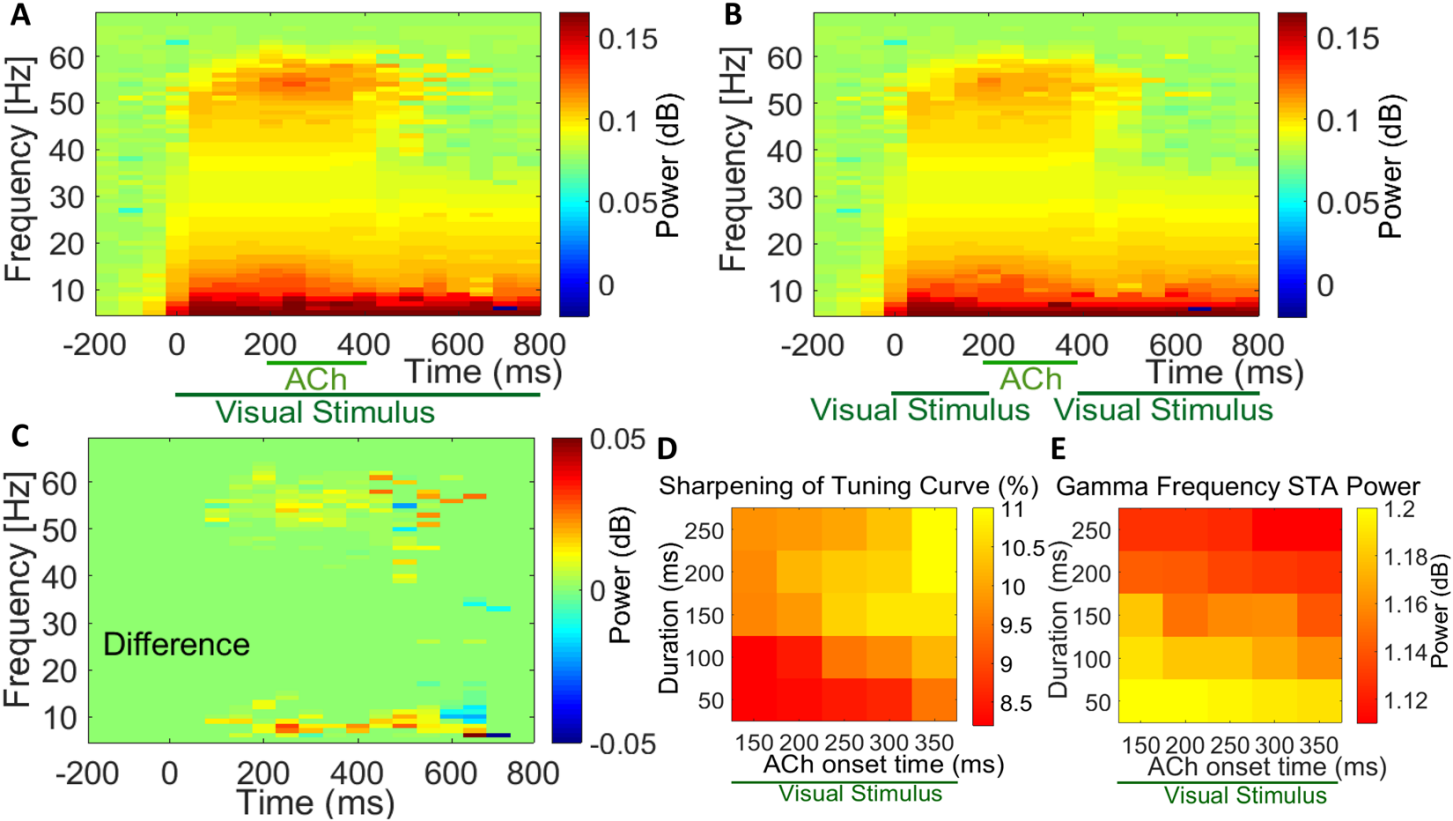
Interaction of Acetylcholine Application Relative to Visual Stimulation and with Different Onset Times and Duration of Administration on STA LFP Frequency Spectra. (**A**) Before cholinergic modulation power at high frequencies is clearly modulated by spiking activity whereas pairing of cholinergic modulation with visual stimulation, caused a significant decrease in gamma frequency power. Visual stimulation lasted 800 ms, shown on the abscissa. (**B**) as (**A**), ACh was applied in the absence of visual stimuli which leads to gamma oscillations reduction. (**C**) The difference between time frequency power spectra STA LFP, when ACh was applied in the presence and absence of visual stimuli. (**D**) Effects of ACh with different onset application times with respect to different duration of administration on the percent of tuning curve sharpening compared to the baseline and (**E**) the gamma frequency power.

Timing of cholinergic activation plays a crucial role in its effect on sensory coding^17^. But, to the best of our knowledge, there has been no modeling studies of the temporal dynamics of cholinergic impact on sensory coding. In this experiment, we investigated the effects of cholinergic modulation onset and duration on the networks’ response synchronization. Our result reveals that applications of acetylcholine in various onset times relative to the stimulus onset and various durations significantly affects the gamma rhythmic activity. In this experiment, time frequency power spectra showed the highest values at low frequency (4–12 Hz) and another peak in the gamma frequency range (30–50 Hz) before the beginning of acetylcholine administration. The power of gamma and low frequency oscillations was significantly reduced by 31% and 6%, respectively (Fig. 6A; time windows 100 *ms*; Wilcoxon signed rank test; *P* < 0.05). Comparison of time frequency power spectra STA LFP when ACh was applied in the absence of visual stimuli revealed that ACh application alone substantially reduced gamma and low frequency oscillations by 23% and 4.3%, respectively (Fig. 6B; time windows 100 *ms*, Wilcoxon signed rank test; *P* < 0.001). During ACh application the oscillatory activities changed dynamically. LFP responses exhibited occasionally transient increase of power in the gamma-frequency range which was followed by a decrease. This was significant for both responses obtained in the presence (Fig. 6A; *P* < 0.007) and absence (Fig. 6B; *P* < 0.007) of visual stimuli during ACh application. We found a significant reduction of gamma activity after combined visual stimulation and cholinergic modulation. There was a significant difference between time frequency power spectra STA LFP when ACh was applied in the presence and absence of visual stimuli (Fig. 6C; *P* < 0.007). Consequently, we found a similar phenomenon with or without pairing of cholinergic modulation with the visual stimulation. We observed lasting activation of different frequencies after termination of the cholinergic input. The continuation of frequency modulation could be due to synaptic delays (>100 *ms*) used in our network model.

In addition, we examined the effects of cholinergic activation onset time and duration on the sharpening of tuning curve and the power of gamma frequency range (Fig. 6D,E). It has been shown that the phasic changes in the cholinergic activation requires modulatory effects sufficiently rapid to increase and decrease within the time course of 100–300 ms^53^. Therefore, we aimed to provide phasic changes in the cholinergic modulatory effects within our network model. These data suggest that application of acetylcholine in various onset time and different durations influenced the sharpening of tuning curve and LFP gamma oscillation. These changes were significant compare to the baseline values (Fig. 6D,E; Wilcoxon signed rank test; *P* < 0.05). Cholinergic modulation with longer duration of administration lead to increased sharpening of orientation tuning curves and decreased LFP gamma power. On the other hand, delayed ACh onset time caused sharper tuning curves and lower LFP gamma power.

## Discussion

In this study, we have proposed a physiologically plausible network model to investigate the mechanisms underlying the influence of acetylcholine (ACh) on the primary visual cortex (V1) neural activity. The principal features of our model are as follows; (i) it is designed based on macaque monkey V1 layer 4C anatomy and physiology data; (ii) It took advantage of V1 cortical connectivity and structure and ring models coordinates^54^, in which coordinate labels were the preferred orientation; (iii) It used spiking neural network and recruited membrane potential synaptic conductance rather than firing rates to drive local field potentials (LFPs); (iv) as a large-scale network model it was constructed of point neurons rather than compartmental models.

In the presence of cholinergic modulation our neuronal network model clearly showed: (i) sharpening of orientation selectivity; (ii) decreased gamma synchrony and spike field coherence; (iii) reduced noise correlation and neural response variability; (iv) predicted a sharpening near the orientation preference; (v) correlation between cholinergic activation onset and duration with network response synchronization and neurons orientation selectivity; (vi) since we used m-current to model cholinergic activation the correlation between the degree of orientation selectivity and the cholinergic activation was mediated by muscarinic receptors; (vii) enhanced inhibitory release in the network that suggests the augmentation of inhibitory tone during ACh release; (viii) role of baseline activity on cholinergic-contingent changes of orientation tuning. Some of these predictions have not been tested experimentally before (see below). Thus, our model provides a computational framework for future experiments.

Modeling studies have been used to replicate the experimental findings and to predict aspects of cholinergic influence that are not presently clear by experimental research. For example, to our knowledge, in the only other modeling study that has examined the role of cholinergic activation on V1 sensory processing Deco and Thiele 2011 investigated the contribution of acetylcholine in feedback-mediated attentional modulation in a network model^49^. In our model, we used a different pattern of cortico-cortical connectivity and architectures to replicate natural V1 anatomy. Specifically, consistent with Deco and Thiele 2011 report, we have found that cholinergic system influences intracortical interactions probably by increasing inhibitory drive. But, in addition, we have examined several important aspects of cholinergic effects in V1 that are novel and have not been examined before. Namely, we have shown significant sharpening in V1 neurons’ orientation selectivity, desynchronization of LFP gamma power and spike-field coherence, decreased response variability, the impact of the onset time and duration of cholinergic activation on its sensory coding effects and the role of baseline modulation on the effects of these cholinergic mediated changes. These new findings shed light on how cholinergic activation improves sensory encoding and discrimination at both the single unit and the neural population levels. Moreover, there is an important difference between the pattern of cortico-cortical connectivity and the network model architecture in our model and Deco and Thiele 2011 model. They used a recurrent network model, whereas we used a biologically plausible ring model that is orientation specific. Our model structure is highly consistent with the anatomy of V1 and their role in the emergence of orientation selectivity. In particular, in our model, the angular modulation of the intracortical interactions (Eq. 12) is consistent with the dendritic and axonal branching and arborization and spatial distributions in macaque primary V1^55^.

Attentional modulation is accompanied by an increase in cortical ACh release suggesting that the augmentation of attentional demand is associated with increase in cortical ACh release^44,45^. The enhancement of cortical ACh release observed in attentional tasks indicates a key role of cholinergic modulation for attentional functions^56–59^ and presents a direct relationship between attentional modulation and cortical ACh release^60^. ACh, as a powerful cholinergic neuromodulator, effects behavior and influences the neural systems at cellular and circuit levels^61^. Particularly, cholinergic activation via muscarinic receptors improves neuronal excitability and enhances stimulus stability and abstract representations^62^. In rodents, the cholinergic system is believed to be part of the central mechanism that mediates attention^63^. Moreover, depletion of ACh in cortical areas by disrupting cholinergic fibers results in diminished attentional impact^12^. The attentional deficit seen in Alzheimer’s disease arises from cholinergic dysfunction^64^. Herrero *et al*. demonstrated that ACh application enhances attentional modulation in V1^10^.

Similar to all of the other modeling studies of the role of cholinergic system in attention the link between our model and biological attentional processes is made by showing similarity of attention contingent neurophysiological events with the performance and outcome of the model. Specifically, our findings provide new modeling evidence that cholinergic system may be used by attentional mechanisms to substantially scale and sharpen stimulus selectivity, induce high desynchronize gamma power and spike field coherence and transiently change correlation structure of the neural network to improve sensory discrimination. In addition, similar to the empirical evidence that show attention can exert its effect by modulating baseline activity^65–68^ our model, for the first time, provides a modeling framework that explains how attention use baseline activity modulation to enhance the effects of cholinergic activation on sensory encoding.

Similar to the effects of attention, cortical iontophoresis of ACh alters neural orientation tuning and direction selectivity. Although the reported effects are variable between neurons and across studies^21,24,25^. Consistently, ACh application and nucleus basalis stimulation increase orientation selectivity in cat visual cortex^21,22^. ACh enhances the amplitude of evoked sensory responses and performs a filtering role through enhancing relevant inputs while suppressing weak sensory inputs causing a sharpening of the neurons tuning curves in sensory cortex^30,69^. While our modeling results are consistent with these experimental studies, it should be noted that some studies have failed to replicate the effect of ACh on tuning curve sharpening^24^.

There are many similarities between the effects of ACh observed in our model and the experimental studies in which animals are engaged in attention-demanding tasks^63,70^. For example, some studies in cat V1 have demonstrated that application of ACh leads to a sharpening of tuning curves^21,22^ which imitates attentional effects^1,8,9,71,72^. But it should be noted that some other studies have failed to replicate this effect^24,25^. The reason for these conflicting results is not clear. Our data is consistent with the studies that have shown attention-dependent scaling and sharpening of neuronal tuning in the ventral visual pathway including V1^7,71,72^. Consistently, other experiments that have found similar attention-dependent sharpening in the orientation tuning properties of monkey V1 neurons^73^. Attention enhances the firing rates when stimuli are presented primarily at or near the peak of the orientation tuning of V1 neurons^71^. But, it should be noted that contradictory to these results other studies investigating the effects of attention on stimulus orientation tuning curve of monkey V1 have found that attention scales the neural orientation tuning without systematically changing its width^74^.

Several experimental studies have shown that enhancement of baseline activity influences neuronal excitability, sensory information coding and behavioral performances^51,67,68,75–77^. Changes in the baseline activity may reflect top-down bias signals and can provide advantage for processing the attended stimuli over unattended stimuli^67^. Attentional task-dependent elevation of the baseline neural firing rates has been revealed in many fMRI and electrophysiology studies^65–68^. Our current modeling study show that the effects of cholinergic activation is enhanced in high baseline activity condition. These results suggest that top-down anticipatory signal that modulate baseline activity can facilitate the influence of cholinergic input in the visual cortex and improve its effect on the sensory coding properties of visual neurons.

It has been suggested that cortical orientation tuning profile of V1 neurons are mediated by thalamocortical connections and by the contribution of inhibitory intracortical connections^26,27^. Cholinergic modulation controls synaptic efficacy of the thalamocortical and intracortical connections^20,28^. We found that cholinergic modulation, similar to attention mechanism, increased inhibitory drive which can lead to neural response selectivity^13,29,30^. Interestingly, we also found a significant tuning-curve amplitude and gain enhancement, without narrowing of tuning width, when ACh was applied only to the excitatory neurons. Our data indicates that, applying ACh to inhibitory neurons led to sharpening and selectivity of tuning curves, as measured by the width of its orientation-tuning curve, while applying ACh to excitatory neurons only caused gain and amplitude enhancement. These findings are consistent with the experimental evidence for gain modulation by nicotine in macaque V1^48^. Our data are also consistent with the experimental research indicating the importance of intracortical inhibition in shaping orientation selectivity in V1^28,29^. According to these studies, inhibitory neurons are responsible for enhanced orientation selectivity as tuning width is mainly determined by the strength of the inhibition and the tuning of the LGN input. Our model suggests a way to reconcile different proposals about whether cholinergic modulation sharpens tuning curves or scales firing rates. Particularly, in the presence of ACh, our model can represent different effects of attentional modulation, like sharpening of orientation tuning^1,3^ or response gain modulation that increase firing rates^74,78^. Our data suggests that cholinergic modulation regulates and controls neural response gain, scales orientation tuning-curve and mediate an important role in response modulation of macaque V1 4C neurons.

It is still unclear how cholinergic modulation coordinates neuronal network information processing. Thus, we specifically examined coordinated spiking across the neural population. Many researchers have demonstrated that attention reduces neuronal synchrony of gamma oscillation in area V1^31,39^. But attention contingent increase of gamma power is also reported for different parts of the visual cortex^37,51^. Cholinergic activation mimics the attention related gamma modulation in V1. For example, Kalmbach *et al*. have demonstrated that ACh reduces gamma synchronization through optogenetical stimulation which leads to cortical neural desynchronization^61^. Also, electrical stimulation of the basal forebrain nucleus desynchronizes cortical activity^28^. On the other hand, ACh has shown to enhance gamma synchronization in anaesthetized cat visual cortex while the application of the muscarinic antagonist scopolamine leads to the reductions of gamma oscillations power^79^. In our model, in the presence of ACh, we found that gamma power decreased in spike triggered averaging (STA), LFP and spike-field coherence (SFC). These findings are reminiscent of decreased gamma power of STA LFP seen in macaque V1 when attention is directed to the receptive field of the neurons being studied^39^. Attention mediated decrease in gamma power could be due to the enhancement of the inhibitory drive through muscarinic activation in V1. Simultaneous decrease in gamma power and increase in firing rates has been shown in V1^40^. Our modeling finding points to the possibility that ACh reduces gamma power causing enhancement of evoked firing rates by weakening the surround inhibition.

The noise correlation was reduced following ACh application in our network model resulting in improved coding accuracy. This suggests reduced trial-to-trial correlation variability in the presence of ACh and thus more reliable sensory representation. These findings are in line with cognitive functions attributed to the cholinergic modulation^12,33,80^. Neural activity decorrelation has been observed to improve population coding accuracy^81^. Consequently, it leads to enhanced information processing through direct involvement of cholinergic mechanisms which is linked to membrane potential fluctuations in cortical neurons and modulation of inhibition in the cortical networks. We also found that ACh significantly enhanced trial-to-trial response reliability as indicated by our Fano Factor calculations. Our findings are consistent with the studies that found basal forebrain cholinergic activation improves neural coding through enhancing the response reliability of individual neurons and decreasing the correlation between neurons^17,33^. Our findings show that enhanced reliability improved the amount of information that each neuron convey; whereas neuronal correlation reduction reduced the redundancy of information in the pool of neurons. Both of these changes highly improved efficiency of sensory information processing. These findings suggest that cholinergic modulation can dynamically regulate cortical coding of sensory inputs which is compatible with the effects of attention on visual neurons^31,32^. Particularly, it has been shown that modulation of network correlation had a significant influence on the attentional function improvement^32,82^. These findings are consistent with the predictions of the normalization model of attention that characterizes how visual attention scales neural responses in the visual cortex^8,82–84^.

Different levels of attention-contingent cholinergic activation have been reported: 120–140%^85^, 150–200%^86^, 140%^87,88^, 210%^89^ and 250–275%^90^. Microdialysis studies found that such enhancement of cortical ACh release is associated with attentional performance augmentation suggesting that cholinergic modulation is a core component of the circuitry that mediates attentional function^86,91^. Consistent with the findings that point to a relation between the level of ACh release and its impact on sensory information processing, a relationship between the degree of damage in the basal forebrain with decreased cortical ACh release and impaired attentional function has been reported^92–97^. Based on these evidences, we used two levels of cholinergic activation using different levels of slow voltage-dependent potassium currents and thalamo-cortical input current. We found that higher concentrations of acetylcholine resulted in narrower and sharper orientation tuning functions, more desynchronization state of LFP gamma power, more decorrelation and higher reliability of neural sensory encoding. These effects are presumably implemented by influencing intracortical interactions and by increasing inhibitory drive within the network^17,33,36,61,98^.

It is possible that the impact of cholinergic modulation depends on the onset timing and duration of ACh application. We tested this possibility by applying the cholinergic input across different onset times and durations. Application of ACh in various onset times relative to the stimulus onset and various durations significantly affected the gamma rhythmic activity and neural orientation selectivity. Cholinergic modulation with longer duration of administration lead to enhance orientation selectivity and reduced LFP gamma power. Also, delayed application of ACh compared to the stimulus onset time, resulted in sharper tuning curves and lower LFP gamma power.

Our network model was constructed from point neurons model with leaky integrate and fire dynamics^99,100^. This is the most popular neuronal model that captures important neuronal properties and widely used for simulating realistic neuronal circuits. We also used m1-type muscarinic receptor through inhibitory neurons^49,101–103^ and nicotinic receptors that are well expressed in the thalamocortical-recipient layer 4C of macaque V1^48,104^. We acknowledge that taking into account the diversity of cell-types in any modeling studies of complex cortical network interactions is vitally important. But, because of the complex nature of the cell and receptor subtypes and due to the lack of sufficient empirical information about their organization and function very few studies have comprehensively included all of the network components in their modeling experiments. Our model has indeed provided new information about the cholinergic system effects on sensory coding and thus has provided a better ground work for future studies that take into account the diversity of the cell and receptor subtypes.

In summary, we propose a computational approach to understand the functional role of cholinergic modulation in the primary visual cortical network and how they might induce specific behaviorally relevant changes. We show increased visually driven cortical firing rates, sharpened stimulus orientation tuning curves, desynchronized LFPs, decreased noise correlation and improved neuronal response reliability. These changes have been reported in primates during selective visual attention. Our proposed model improves our understanding of the cholinergic role in the neural origin in the ring model structure. At the circuit level, cholinergic modulation controls the frequency content and neural cortical oscillations and has network effects relevant to sensory processing associated with attentional processing. Overall, our model presents effects similar to those of attention, and suggests that cholinergic modulations may mediate attentional modulation in V1 layer 4C.

## Methods

### Network structure

**Figure 7:**
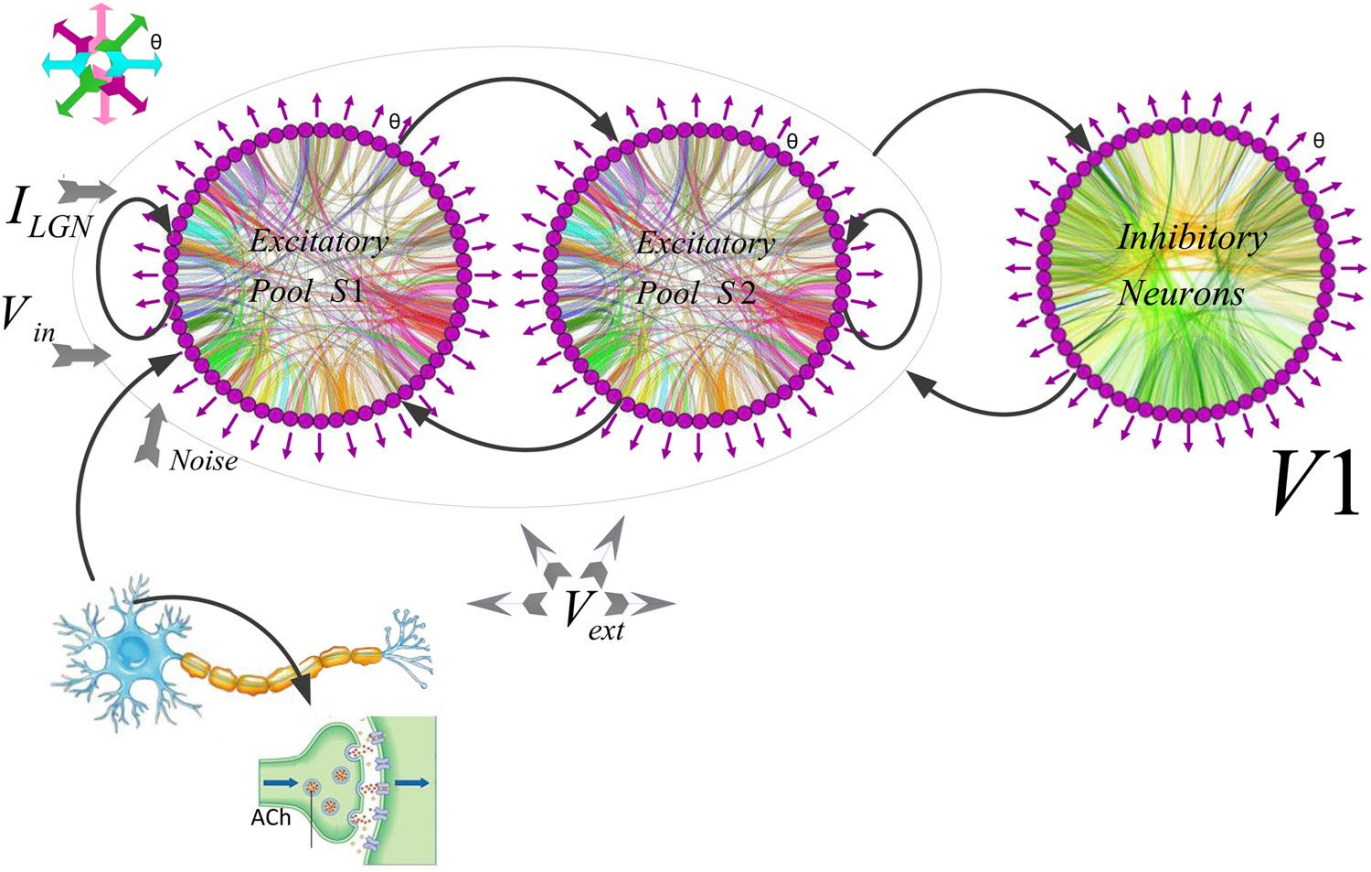
Model Architecture and Representation. The network module consists of excitatory and inhibitory neurons. Each circle is a population of Neurons. The excitatory neurons are organized in two groups of selective (S1 and S2) and a group of inhibitory neurons. All the neurons get an input (νext) which simulates the spontaneous activity in the surrounding cerebral cortex. The neurons in the first excitatory group receive the input encoding the stimulus (νin). ILGN represented feed-forward excitatory input from LGN to the network. The orientation preference *θ* of each neuron was uniformly distributed between −90 and 90 degrees. The distribution’s orientation at angle *θ* in the visual field sets the orientation preference that indicated via purple arrows schematically. The color curves in each circle represents the Gaussian connections. Neurons are connected through recurrent connections mediated the excitatory and inhibitory inputs to neurons through connections with a Mexican hat profile (not all connections shown). Between-pool connections (weights from the inhibitory to the excitatory pools) and recurrent connections present with black arrows. See Methods for details. NetworkX Simulator^123^ was used to generate the figure.

We constructed a computational recurrent network model, a spike-based version of the ring model of layer 4C of the primary visual cortex (V1)^27,54^ which is shown schematically in Fig. 7. The model includes two groups of excitatory and one group of inhibitory neurons. The neurons in each group are specified by the input they receive and high mutual connection strength. Each excitatory group (S1 and S2) represented a separate location in cortical space encoding two non-overlapping receptive fields (RF). Each individual excitatory pool was driven by various kinds of input. The neurons in the first excitatory pool (S1) received external inputs encoding stimulus-specific information but no such input was introduced to the inhibitory neural pool. This is similar to the conditions in which stimulus activates the RF of a specific orientation column in V1 but could have suppressive effects through inhibitory interneurons over neural pools that do not receive direct excitatory input from incoming afferent activity. In addition, our model has the advantage to provide access to the spikes and LFPs of each selective excitatory pool which can provide the opportunity to investigate the direct as well as indirect population responses. The excitatory groups were connected to a group of inhibitory neurons that mediated inhibition in the network. There was a competition between the two excitatory groups using a shared feedback inhibition from the inhibitory neurons. Our model included the essential cortical mechanisms and our analyses enable comparison between the related neurophysiological data and the behavior of our model. Our model consists of 1000 orientation-selective pyramidal neurons and 250 orientation-selective interneurons V1 layer 4C, respecting the 4:1 proportion observed in the cortex^105^.

### Neuron dynamics

We implemented pyramidal (*N*_*E*_) and inhibitory (*N*_*I*_) units as leaky integrate-and-fire neurons (LIF) that were characterized by their membrane capacitance *C*_*m*_, leak conductance *g*_*L*_, conductance of the slow potassium current *g*_*KS*_, fixed voltage threshold *V*_*th*_ for spike inception. Then a spike a refractory time period *r*_*ref*_ and a reset membrane potential of *V*_*reset*_ are considered. All of the parameter values used in our simulation are listed in Table 1. Behavior of our modeled neurons were evaluated by voltage-gated conductance equations with angular dependence that evolved in time as depicted in the following formula:

1 $${C}_{m}\frac{dV(\theta ,t)}{dt}=-\,{g}_{L}(V(\theta ,t)-{V}_{L})+{g}_{KS}(V(\theta ,t)-{V}_{L})-{I}_{syn}(\theta ,t),$$

**Table 1:** Parameter Values for the Network Model of Ring Spiking Neurons.

All neurons		
**Symbol**	**Description**	**Value**
*θ*	Neuron’s Preferred Orientation	(−90,90)degree
*θ_stimulus*	Test Orientation	(−90,90)degree
*V*_*L*_	Leak Reversal Potential	−70.0 mV
*V*_*th*_	Threshold Potential	−50.0 mV
*V*_*reset*_	Reset Potential	−55.0 mV
**Excitatory Neurons**		
*N*_*E*_	Number of Pyramidal Neurons	1000
*C*_*m*_	Membrane Capacitance	0.5 nF
*g*_*LE*_	Total Leak Conductance	25.0 nS
*τ*_*rE*_	Refractory Time	2.0 mS
**Inhibitory Neurons**		
*N*_*I*_	Number of Interneurons	250
*C*_*m*_	Membrane Capacitance	0.2nF
*g*_*LI*_	Leak Reversal Potential	20.0 nS
*τ*_*rI*_	Refractory Time	1.0 ms
*V*_*I*_	Potential	0.0 mV
**AMPA Receptor**		
*τ*_*AMPA*_	Exponential Decay Time Constant of AMPA currents	2.0 mS
*E*_*AMPA*_	Synaptic Reversal Potential	0.0 mV
*g*_*ext−E*_	Conductance from External to Pyramidal Neurons	2.1 nS
*g*_*ext−I*_	Conductance from External to Interneuron Neurons	1.62 nS
*g*_*AMPA−E*_	Conductance from Pyramidal to Interneuron Neurons	0.04 nS
*g*_*AMPA−E*_	Conductance from Pyramidal to Pyramidal Neurons	0.05 nS
**GABA Receptor**		
*τ*_*GABA*_	Exponential Decay Time Constant of GABA Currents	5.0 ms
*E*_*GABA*_	Synaptic Reversal Potential	−70.0 mV
*g*_*GABA–I*_	Conductance to Interneuron Neurons	1.3 nS
*g*_*GABA–E*_	Conductance to Pyramidal Neurons	1.0 nS
**NMDA Receptor**		
*τ*_*NMDA*_	Decay Time of NMDA Currents of NMDA Currents	100.0 ms
*E*_*NMDA*_	Synaptic Reversal Potential	0.0 mV
*g*_*NMDA–E*_	Conductance to Pyramidal Neurons	0.165 nS
*g*_*NMDA–I*_	Conductance to Interneuron Neurons	0.13 nS
*τ*_*X*_	Controls The Rise Time of NMDAR Channels	2.0
*α*	Controls The Saturation Properties of NMDA Channels	0.5 kHz
*a*	Control The Voltage Dependence of NMDA Channel	0.062 mV
*b*	Control The Voltage Dependence of NMDA Channel ([Mg2+] = 1 mM	1/3.57

The orientation preference *θ* of each neuron was uniformly distributed between −90 and 90 degrees. The distribution’s orientation at angle *θ* determines the orientation preference in the visual field and are schematically shown by purple arrows in Fig. 7.

Neurons were connected using connections with Mexican hat function through recurrent connections driven by the excitatory and inhibitory inputs (Fig. 7; not all connections shown). In line with the electrophysiological studies, both excitatory and inhibitory connections were strongest for neurons with similar preferred orientation. These values decreased as the difference between the preferred orientations of the neurons increased. The inhibitory connectivity was wider than the excitatory ones and the sum of interaction among neurons had a profile similar to Mexican hat. This pattern of interaction can increase the small orientation bias which is conveyed by the feed-forward input that contribute to the V1 orientation tuning curves (Table 1). We exposed the excitatory and inhibitory neurons to an external input (*v*_*ext*_ = 2.4 kHz). They were modeled as a Poisson spike train fired at 3 Hz. These properties are consistent with neural spontaneous activity of the cerebral cortical neurons^93^.

### Synapses

We used different excitatory and inhibitory receptors to induce synaptic currents *I*_*Syn*_ in the neurons. The recurrent excitatory postsynaptic EPSPs had two components and was mediated by AMPA and NMDA receptors that were activated by glutamate. External synaptic inputs conveyed the information to the network about stimuli, mediated external top–down interaction and background noise coming from the spontaneous activity outside the neural network. In our simulations, external EPSCs were induced particularly by AMPA receptors on pyramidal and interneurons. We also assumed that recurrent excitation is largely induced by the NMDA receptors. Inhibitory GABAergic GABAA receptors on interneurons and pyramidal neurons yield the IPSPs. We considered that the NMDA currents have a voltage dependence that is controlled by the extracellular magnesium concentration [*Mg*^2+^] = 1 mM. AMPA and NMDA receptors have different time constants: AMPA decayed fast whereas NMDA decayed slowly^106^. The decay constant for GABA was defined between those two values^107^. These decay constants specify the oscillation frequency of the network (see below).

The synaptic current was given by the sum of glutamergic AMPA, *I*_*AMPA,rec*_ and NMDA, *I*_*NMDA,rec*_ which mediated recurrent excitatory currents, inhibitory GABAergic current *I*_*GABA,rec*_ and *I*_*AMPA,exc*_ which mediated external excitatory current and *I*_*ffLGN*_ feed-forward excitatory input from LGN current. The total synaptic current was given by the sum:

2 $$\begin{array}{rcl}{I}_{syn}(\theta ,t) & = & {I}_{AMPA,ext}(\theta ,t)+{I}_{AMPA,rec}(\theta ,t)+{I}_{ffLGN}(\theta ,{\theta }_{stim})+{I}_{NMDA,rec}(\theta ,t)\\  & + & {I}_{GABA,rec}(\theta ,t)+{I}_{drive}(\theta ,t)\end{array}$$

The currents were defined by:

3 $${I}_{AMPA,ext}(\theta ,t)={g}_{AMPA,ext}(V(\theta ,t)-{V}_{E})\mathop{\sum }\limits_{j=1}^{{N}_{ext}}{{s}_{j}}^{AMPA,ext}(\theta ,t)$$

4 $${I}_{AMPA,rec}(\theta ,t)={g}_{AMPA,rec}(V(\theta ,t)-{V}_{E})\mathop{\sum }\limits_{j=1}^{{N}_{E}}{w}_{j}{s}_{j}^{AMPA,rec}(\theta ,t)$$

5 $${I}_{GABA,rec}(\theta ,t)={g}_{GABA}(V(\theta ,t)-{V}_{I})\mathop{\sum }\limits_{j=1}^{{N}_{I}}{s}_{j}^{GABA}(\theta ,t)$$

6 $${I}_{NMDA,rec}(\theta ,t)=\frac{{g}_{NMDA}(V(\theta ,t)-{V}_{E})}{(1+[M{g}^{2+}]\exp (\,-\,0.062V(t))/3.57)}\mathop{\sum }\limits_{j=1}^{{N}_{E}}{w}_{j}{s}_{j}^{NMDA}(\theta ,t)$$

The weights *w*_*j*_ present the structured excitatory recurrent connections, the sum over *j* presents an aggregation over the synapses constituted by presynaptic neuron *j*. The recurrent excitation was mediated by the NMDA receptors and the network was dominated by recurrent inhibition. The gating variables of open channels *S* are characterized as follow equation as well as the channels equations which are represented in the following. Note that, sum over *k* presents a sum over spikes *δ*(*t*) of the presynaptic neuron *j* at time of ${t}_{j}^{k}$.

7 $$\frac{d{s}_{j}^{AMPA}(\theta ,t)}{dt}=-\,\frac{{s}_{j}^{AMPA}(\theta ,t)}{{\tau }_{AMPA}}+\sum _{k}\delta (t-{t}_{j}^{k})$$

8 $$\frac{d{s}_{j}^{NMDA}(\theta ,t)}{dt}=-\,\frac{{s}_{j}^{NMDA}(\theta ,t)}{{\tau }_{NMDA,decay}}+\alpha {x}_{j}((\theta ,t)(1-{s}_{j}^{NMDA}(\theta ,t)))$$

9 $$\frac{d{x}_{j}(\theta ,t)}{dt}=-\,\frac{{x}_{j}(\theta ,t)}{{\tau }_{NMDA,rise}}+\sum _{k}\delta (t-{t}_{j}^{k})$$

10 $$\frac{d{s}_{j}^{GABA}(\theta ,t)}{dt}=-\,\frac{{s}_{j}^{GABA}(\theta ,t)}{{\tau }_{GABA}}+\sum _{k}\delta (t-{t}_{j}^{k})$$

Note that the parameters, including synaptic conductances, used in our model were selected for two main reasons. First, we used conductance values based on the experimentally measured parameters^108^. Also, the values for voltage potentials, membrane capacitances, time constants and all other parameters were selected according to the values previously used in modeling studies^109^ which are used based on experimentally measured values^108^. Second, we used the properties of the receptor activations that is captured by plausible kinetics scheme and experimentally measured procedures which provide a good description of ionic currents^108–110^. The parameters were based on the measurements of miniature synaptic currents in the neocortex^111^ and the analysis^112^ that lead to estimation of AMPA conductance around 0.35 to 1nS, and the conductance of NMDA-mediated currents which is a fraction of AMPA channels: between 3 to 62%^113^ leading to NMDA conductance estimates around 0.01 to 0.6nS. These parameters have been previously tested and optimized^109^.

### Network connections

The input *I*_*ffLGN*_ presented feed-forward excitatory input from lateral ganglion nucleus (LGN) to network which was considered as a Gaussian profile with periodic boundary conditions^114^:

11 $${I}_{ffLGN}(\theta ,{\theta }_{stim})={J}_{LGN}C\,Exp[\,-\,{(\theta -{\theta }_{stim})}^{2}/2{\sigma }_{LGN}^{2}]$$

Where *σ*_*LGN*_ presents the Gaussian width of stimulus profile and *J*_*LGN*_ its amplitude. Each individual neuron receives inputs from all other neurons through structured synaptic weights. Recurrent connections mediated the excitatory and inhibitory inputs to the neurons according to the equations where *θ*_*i*_ and *θ*_*j*_ represented the presynaptic and postsynaptic neurons, respectively^115^. We used the following periodic function for the connection probabilities:

12 $$K(\theta )=C[COS(2\theta )+1]$$

The function *K*(*θ*) was normalized to the constant *C*. Therefore, the sum of connections from any neuron to the rest of the population equals to: 1/*C* = ∑_*i*_|*K*(*θ*_*i*_)|. The connections were strongest among the neurons with the same preferred orientation and decreased with increasing the difference between the preferred orientations of the neurons. Excitatory and inhibitory connections and interaction lead to a center-surround or Mexican-hat profile.

We considered a correlated construction in the LGN input to V1 layer 4C and designed a ring model structure. Receiving feedforward LGN input *I*_*ffLGN*_ set up an orientation preference at the center of the pattern. The differences between neuron’s selectivity emerged through their connection and non-selective inhibition like a function of orientation^116^. We have mapped the neurons’ orientation preferences using radial spoke as illustrated in Fig. 7. The connection weights were described as adjustable parameters in the model. Using this selection of synaptic strengths and configuration structure made the network stable with highly orientation tuned neurons^54,114,117,118^. It also led to high membrane conductance during stimulation.

### Simulation of the presence of cholinergic neuromodulation

Acetylcholine (ACh) is a cortical neuromodulator involved in cognitive control^10,12,49,119^. *In-vitro* and *in vivo* studies suggest that ACh influence the flow of information in the cortical areas. This function is mediated by reducing the efficacy of cortical synapses through activation of muscarinic receptors^13^ and enhancing the efficacy of feed-forward, thalamocortical input^20^ by influencing nicotinic receptors. Experimental studies suggest that cholinergic afferents target GABAergic interneurons; consequently, activation of these inhibitory neurons suppress cholinergic release^29,48,120^. These findings are supported by other reports showing that inhibitory interneurons in macaque V1 strongly express muscarinic receptors^120,121^. Although, other studies show that some subtypes of muscarinic receptors are expressed in both excitatory and inhibitory neurons^121^. Both ACh receptors are expressed at significant levels within layer 4C which is the principal thalamocortical recipient part of macaque V1^48^.

Therefore, in this study, cholinergic modulation is modeled with inhibitory interneurons and synaptic transmission of inhibitory connections by reducing lateral interactions of muscarinic presynaptic receptors and by enhancing the inhibitory drive. In addition, Krnjevik *et al*. (1971) attributed the effects of cholinergic modulation to the blocking of K + conductance through the activation of muscarinic receptors using intracellular recordings from cortical neurons^122^. Also, voltage clamp recording by Brown and Adams (1980) of muscarinic depolarizations in frog sympathetic neurons illustrated that the effects are mediated largely by a decrease in a voltage and time dependent potassium K + currents^103^. In our network, the presence of m1-type muscarinic ACh and its effects were modeled by modulating the maximum conductance of the muscarinic potassium M-current *g*_*KS*_ of a slow, low threshold K + set to zero to simulate high ACh concentration and set to1.5 *μS*/*cm*^2^ to simulate low ACh concentration^43,101,103^.

We defined *I*_*drive*_ as an external current with Gaussian distribution across neurons in our network through a variance that caused a spread of 1 Hz in the intrinsic neuronal frequencies for both high and low concentrations of ACh. Therefore, we the mean distribution of *I*_*drive*_ values were 0.08 *μA*/*cm*^2^ for high ACh and 1.30 *μA*/*cm*^2^ for low ACh. Also, we considered cholinergic modulation mediated by nicotinic receptors through feed-forward excitatory input current (thalamic afferent fibers) arriving from LGN to the network^20,48,49^. We investigated nicotinic effects through perturbation thalamocortical current *I*_*ffLGN*_, by changing cholinergic activity and measured its impact on the behavior of our model. We considered two levels of ACh with the aim of investigating the consequence of cholinergic modulation of various potassium and leak currents on the model behavior^43^. Note that utilizing different levels of ACh was essential to consider different firing thresholds and frequency-current. To achieve stable baseline condition in every trial we simulated the network without any input till the neural activity stabilized. Then a stimulus with orientation *θ*_*stim*_ was presented for 500 *ms* duration. The response to stimulus was tested using the network activity equilibration. The activity at the end of this time was related to a single trial during 100 *ms* time windows. For the numerical integration of all neural dynamics equations with an integration time step *dt* = 0.02 *ms* the simulations were run in Brian version 1, under Python 2.7 and were tested in Matlab software 2010b. NetworkX Simulator^123^ was used to generate Fig. 7.

### Data analysis

#### Local field potential (LFP)

In this paper, we simulated LFPs to further reveal network level neural computations in cortical circuits^124,125^. LFP reflects important synaptic processes which cannot be measured by looking at the spiking activity of individual neurons. In our simulations, we used spikes of all of the neurons in different groups and calculated the LFPs as average activity of all the neurons. We used several previously proposed approaches for calculating the LFPs including: averaging the spike rates of neural population; averaging the membrane potentials of all neurons; averaging the incoming synaptic currents to a neuron across the neural population; and finally aggregating the absolute values of synaptic currents. In this paper, all of the computations led to highly similar results thus we only report simulated LFPs obtained by averaging incoming synaptic currents to a neuron across the neural population in our model network.

#### Spike-triggered average (STA)

To measure the neuronal synchronization between spikes and LFPs we used the spike-triggered average (STA)^37^. LFP fluctuations increase during the spike-triggered averaging process when spike times have a reliable temporal relation to the LFP. But if spike times are not temporally locked to the activity of neighboring neurons LFP averages out during STA compilation and yields a flat STA. For the simulation of the STA we first obtained short time windows of ±100 *ms* around each spike time and then cut out the related LFPs segments. STA is the average over all these windows so components of the LFP that are not temporally related to the spikes will be averaged out and are thus not visible in STA results.

We normalized STA values for the spike numbers as it was calculated by summing all LFP segments and then was divided by the number of spikes. To characterize the STA we calculated its power spectrum using Fast Fourier Transformation (FFT) and then the results were normalized by dividing it by the total power in the spectrum^37,49^.

#### Spike-field coherence (SFC)

The spike-field coherence (SFC) calculates phase synchronization between spike times and the LFPs across a range of oscillation frequencies^37^. To quantify the phase synchronization between spikes and the LFP the power spectrum of the STA should be normalize. To do so, we averaged all power spectra of all of the LFP segments. This normalization yields the SFC values. Note that SFC is not contingent on the spike firing rates and the LFP power spectrum. For any frequency, SFC value of one indicates that time of all of the spikes are locked to the phase of the given frequency. On the other hand, SFC values of zero indicates that the time of spikes do not have any relation with the phase of the given LFP frequency.

### Noise correlation

To characterize noise correlation *r*_*sc*_, we computed the Pearson correlation coefficient of the neurons’ responses between each pair and then averaged the correlation coefficients for all potential pairs of neurons^32^. The Pearson correlation coefficient of the spike counts of a pair of neurons to the presentation of the stimulus was calculated as follows:

13 $${r}_{sc}=\frac{E[{N}_{i}{N}_{j}]-E{N}_{i}E{N}_{j}}{{\sigma }_{{N}_{i}}{\sigma }_{{N}_{j}}},$$

where *E* is the expected value, *σ*_*N*_ is the standard deviation of the neuron responses, and *N*_*i*_, *N*_*j*_ are the spike counts of neuron *i* and *j*, respectively. Additionally, for the statistical significance of the experiments, repeated measure two-factor ANOVAs (factor 1: ACh applied/not applied; factor 2: stimulus orientation, bin size 10°) were utilized to analyze the effects of ACh on the network model. Statistical significance was measured using ANOVA and *p* ≤ 0.05 was set as the significancy level.

### Fano factor

Trial-to-trial variability was calculated by the Fano factor (FF) which was defined as the ratio of the variance of the number of spikes across trials (spike counts) divided by the mean of the spike counts^126^. In the Fano factor analysis we used 100 ms time windows and calculated the spike counts within these windows in order to determine whether acetylcholine had an effect on the variability of the spiking rate. Statistical significance was evaluated by permutation tests between the ACh applied in high and low concentration and control condition. The FF was calculated using the following formula:

14 $$FF=\frac{{\rm{variance}}\,({\rm{spike}}\,{\rm{count}})}{{\rm{mean}}\,({\rm{spike}}\,{\rm{count}})},$$

## Supplementary information

Supplementary Information.
