# Supplementary material for: Cholinergic Modulation Promotes Attentional Modulation in Primary Visual Cortex- A Modeling Study
Source: Sci Rep. 2019 Dec 27;9:20186. doi: 10.1038/s41598-019-56608-3 (PMC6934489; doi:10.1038/s41598-019-56608-3)
Supplement: Supplementary file 1 — Supplementary Information. [file 41598_2019_56608_MOESM1_ESM.pdf]

Supplementary Information: Cholinergic Modulation Promotes Attentional Modulation in Primary Visual Cortex- A Modeling Study

Atena Sajedin, Mohammad Bagher Menhaj, Abdol-Hossein Vahabie,
Stefano Panzeri, and Hossein Esteky

Supplementary Figures

Figure S1. Effects of varying the slow potassium conductance upon percentage of power in the gamma-frequency band of the STA LFP. For each value of potassium conductance, we plot the percentage of power in the gamma frequency band. The error bars indicate the 95% confidence intervals.

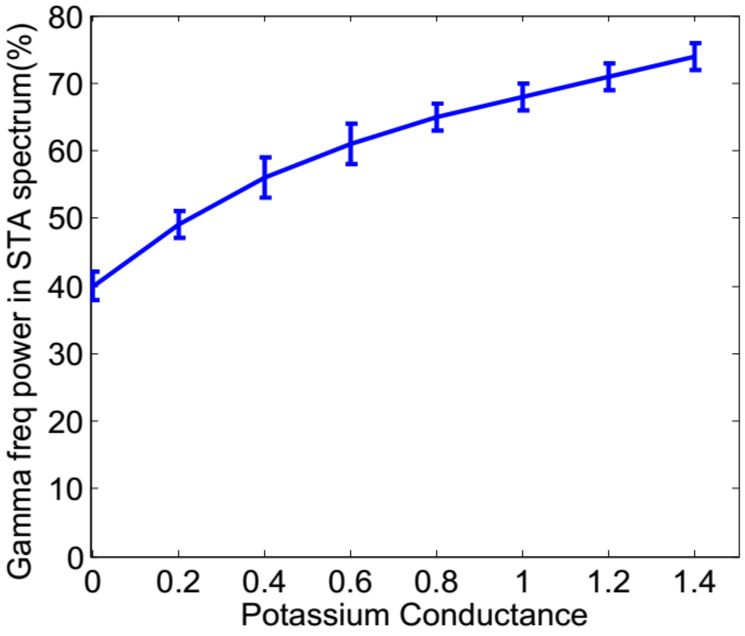

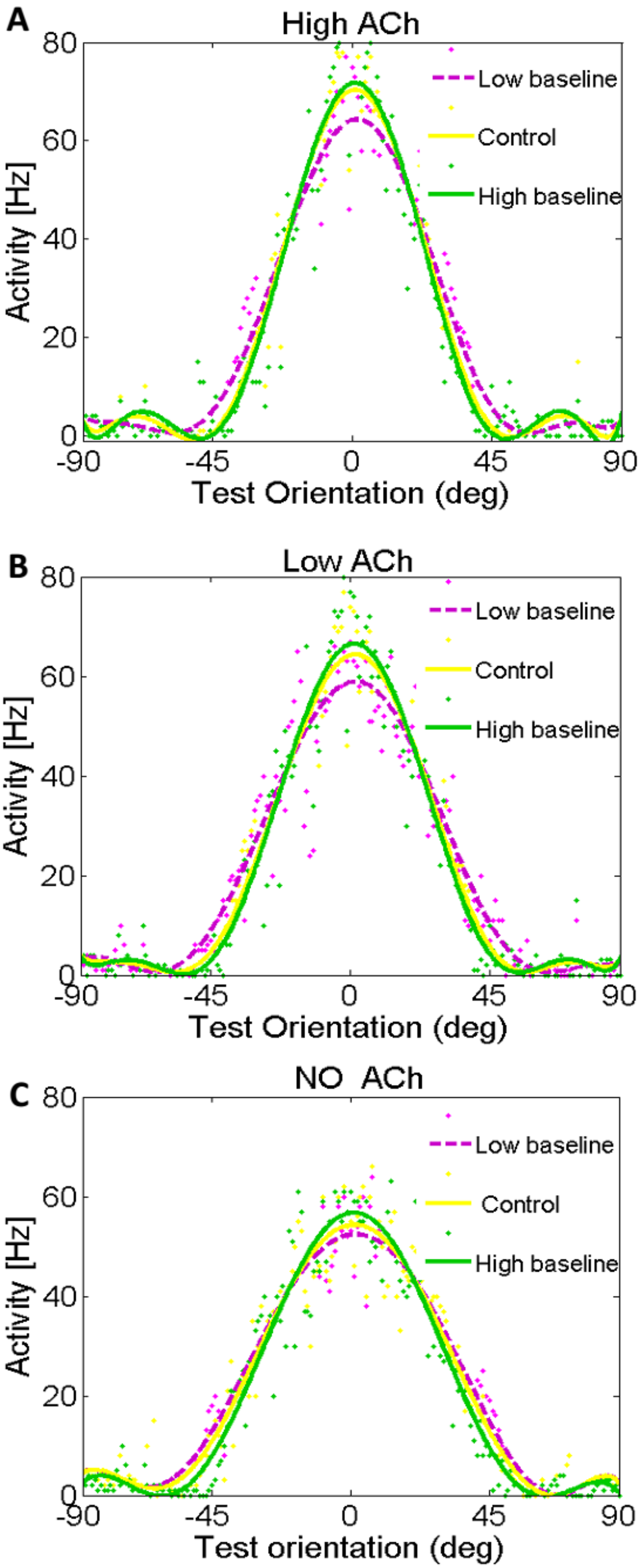

Figure S2. Effect of different levels of ACh application on the tuning curves of the individual neurons, with different levels of baseline activity of the network. (**A**) Applying High level, (**B**) low level of ACh, and control condition (**C**) in different baseline activity strongly alters orientation selectivity of V1 neurons.
